# Incidence and risk factors for rotator cuff retear after arthroscopic rotator cuff repair in middle-aged and older patients: a systematic review and meta-analysis

**DOI:** 10.1186/s12891-026-10337-7

**Published:** 2026-08-10

**Authors:** Hongyu Wang, Zhiruo Jiang, Qingyun Xue, Lei Shi, Fei Wang, Haishi Zheng, Lin Wang

**Affiliations:** 1 Department of Orthopaedics, Beijing Hospital, National Center for Gerontology; National Clinical Research Center for Gerontology; The Key Laboratory of Geriatrics of NHC; Institute of Geriatric Medicine, Chinese Academy of Medical Sciences, NO.1 Da Hua Road, DongDan, Beijing, 100730 People’s Republic of China; 2 Peking University Fifth School of Clinical Medicine, NO.1 Da Hua Road, DongDan, Beijing, 100730 People’s Republic of China

**Keywords:** Rotator cuff tear, Arthroscopic repair, Retear, Risk factor, Meta-analysis

## Abstract

**Background:**

Rotator cuff retear remains a major challenge after arthroscopic rotator cuff repair (ARCR), particularly among middle-aged and older patients. This study aimed to investigate the incidence of retear and identify its associated risk factors in this patient population.

**Methods:**

A single-arm proportion meta-analysis was conducted to pool the overall retear rate among studies reporting the number of retear events, total sample size, and enrollment of > 50 participants. Furthermore, meta-analyses were performed for retear-related risk factors reported in the included studies, from which odds ratios (ORs) could be extracted or calculated. The methodological quality of the included studies was assessed using the Agency for Healthcare Research and Quality (AHRQ) Checklist. Heterogeneity was evaluated using I^2^, and fixed- or random-effects models were selected accordingly. Sensitivity analyses and Egger’s tests were used to assess robustness and publication bias.

**Results:**

In total, 273 studies (147,042 patients) were included in this single-arm proportion meta-analysis. The pooled overall retear rate after ARCR in middle-aged and older patients was 17% (95% confidence interval, 16%-18%). For the risk factor meta-analyses, 35 cohort/case-control studies (12,031 patients) were included. Retear risk was significantly associated with male sex (OR = 1.41), smoking history (OR = 2.21), diabetes mellitus (OR = 2.07), hyperlipidemia (OR = 3.45), greater fatty infiltration (supraspinatus Goutallier grade: OR = 2.70; infraspinatus Goutallier grade: OR = 2.79; global fatty degeneration index: OR = 3.64), and larger tear size (anteroposterior dimension per 1-cm increase: OR = 1.48; mediolateral dimension per 1-cm increase: OR = 1.17; large to massive tear: OR = 2.11).

**Conclusions:**

Retear after ARCR is common in middle-aged and older patients, with an overall incidence of 17%. Retear is closely associated with metabolic comorbidities (diabetes mellitus and hyperlipidemia), male sex, smoking status, tear size, and degree of rotator cuff muscle fatty infiltration. Comprehensive preoperative evaluation incorporating these risk factors may improve risk stratification, guide surgical decision-making, and support the use of adjunctive strategies in high-risk patients to reduce retear rates and optimize outcomes.

**Supplementary Information:**

The online version contains supplementary material available at https://doi.org/10.1186/s12891-026-10337-7.

## Introduction

Rotator cuff tear is one of the most common shoulder disorders in middle-aged and older adults, with prevalence increasing substantially with age [1]. Epidemiologic studies have reported a prevalence of approximately 35% among individuals > 60 years and as high as 46.4% among those > 70 years [2]. Arthroscopic rotator cuff repair (ARCR) has become the standard surgical procedure for rotator cuff tear because of its minimally invasive nature, precision, and faster recovery [3]. However, despite ongoing advances in surgical techniques, anchor materials, and postoperative rehabilitation protocols, rotator cuff retear remains the most common postoperative complication and can substantially impair functional recovery and quality of life [4]. In older patients, retear risk increases with age [5]. Prior reports suggest a marked increase in postoperative retear rates among patients > 70 years [6], which may be attributable to age-related rotator cuff degeneration and poorer tendon quality.

Increasing evidence suggests that rotator cuff retear is associated with recurrent shoulder pain, decreased strength, restricted range of motion, and significantly poorer functional outcomes compared with intact repairs [7, 8]. Retear also increases the risk of revision surgery and health care burden [9], and some patients may ultimately require reverse total shoulder arthroplasty [10]. Therefore, identifying risk factors for rotator cuff retear, performing preoperative risk stratification, and developing individualized treatment strategies are clinically important for improving surgical success and patient outcomes.

The postoperative retear rate reflects the structural success or failure of tendon repair; however, reported retear rates vary widely across studies. Moreover, systematic quantification and bias assessments of retear incidence, specifically in middle-aged and older populations, are limited. Multiple retear-related risk factors have been reported [11, 12], including patient factors (advanced age, male sex, smoking, diabetes mellitus, hyperlipidemia and other metabolic diseases) [13–17], tear characteristics (large or massive tear, involvement of multiple tendons, and degree of tendon retraction) [18], muscle quality (Goutallier grade > 2 fatty infiltration and muscle atrophy) [19], and surgical/management factors (procedure selection, fixation strength, and rehabilitation protocols) [20]. However, most available studies are single-center, small-sample, retrospective analyses with inconsistent findings, and systematic evidence focusing on middle-aged and older patients remains relatively scarce.

Given these considerations, this systematic review and meta-analysis aimed to quantify the overall retear rate after ARCR in middle-aged and older patients using a single-arm proportion meta-analysis and systematically evaluate and quantify the effect sizes of the risk factors associated with retear. This evidence may inform the preoperative risk stratification, perioperative metabolic management, and postoperative rehabilitation strategies.

## Methods

### Study design and registration

This systematic review and meta-analysis was conducted in accordance with the Preferred Reporting Items for Systematic Reviews and Meta-Analyses (PRISMA 2020) statement [21] (Supplementary Material 1). The study protocol was registered a priori in PROSPERO (registration number CRD420251161241).

### Search strategy and study selection

Four English-language electronic databases were systematically searched: the Web of Science (WOS), Cochrane Library, PubMed, and Embase. The search covered inception through October 1, 2025, with no language restrictions. The search strategy combined controlled vocabulary terms (MeSH/Emtree terms) and free-text terms. Core search terms included rotator cuff injury (“rotator cuff injury,” “rotator cuff tear,” “rotator cuff rupture”); arthroscopic surgery (“arthroscopy,” “arthroscopic repair,” “arthroscopic surgical procedure”); target population (“middle aged,” “elderly,” “aged”); and outcomes (“retear,” “rotator cuff retear,” “revision”). Boolean operators “AND” and “OR” were used. The complete search strategy is described in Supplementary Material 2.

**Fig. 1:**
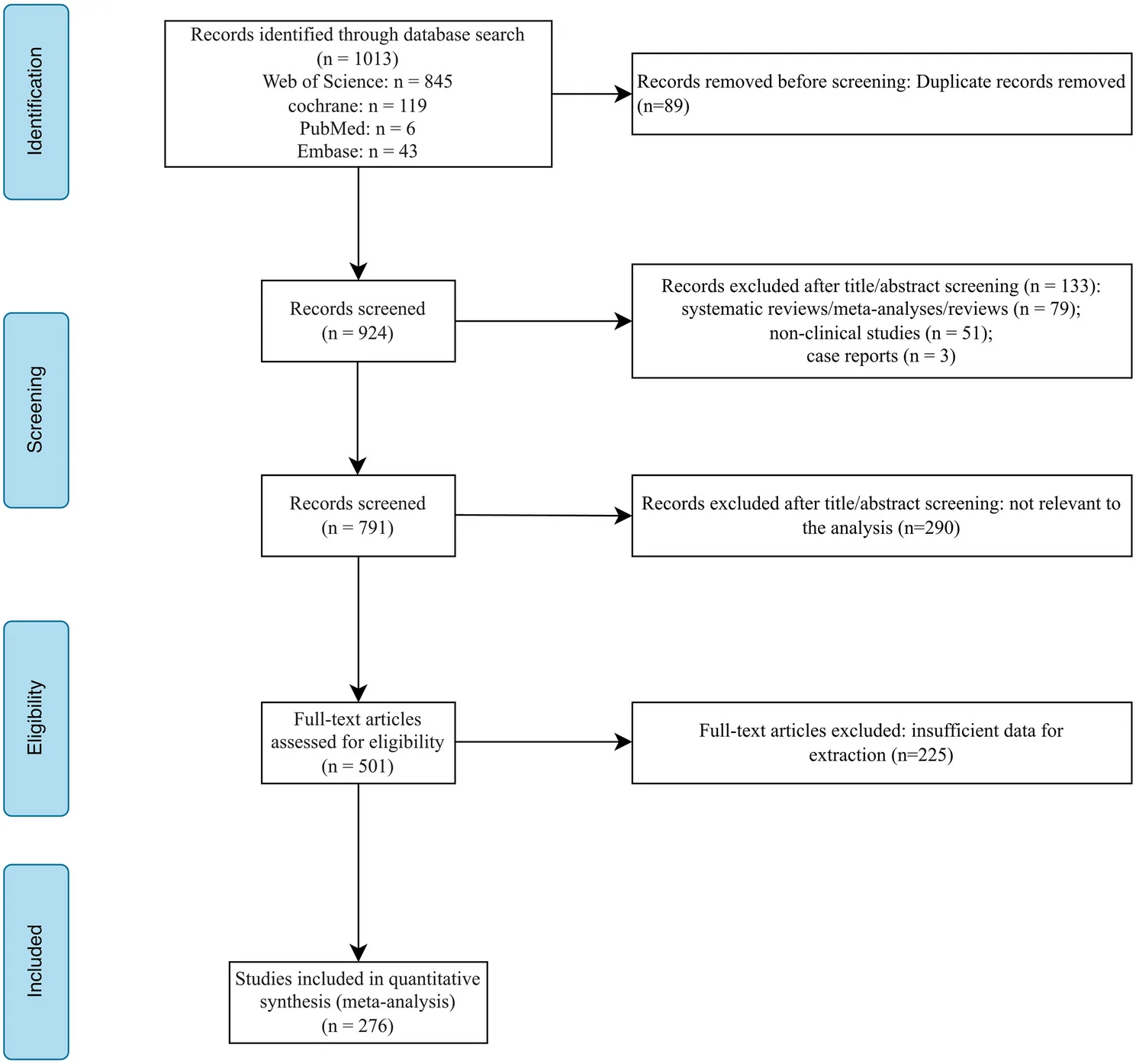
PRISMA 2020 Flow diagram of study selection

Study selection was independently performed by two reviewers (HW, ZJ) trained in systematic review methodology. Duplicates were removed using EndNote X9, followed by title/abstract screening according to pre-specified inclusion and exclusion criteria. The full texts of potentially eligible studies were assessed to determine their final eligibility. Disagreements were resolved through discussion and adjudication by a third reviewer (LW) when necessary. The selection process is presented in the PRISMA 2020 flow diagram (Fig. 1).

### Inclusion and exclusion criteria

1. Inclusion criteria.
  (1) Middle-aged and older patients (age ≥ 45 years) who underwent ARCR [22, 23]. (2) The study analyzed at least 1 risk factor associated with postoperative rotator cuff retear. (3) The primary outcome was postoperative rotator cuff retear, confirmed by objective imaging (magnetic resonance imaging [MRI] or ultrasonography) [24, 25]. Studies are required to clearly report imaging-based criteria, such as definitions of healing and retear. To pool the overall retear rate, studies had to report the extractable number of retear events and total sample size, with a sample size of > 50. (4) Studies included in the pooled analysis of risk factors were required to provide extractable raw data or effect size estimates such as odds ratios (ORs) with 95% confidence intervals (CIs).
2. Exclusion criteria.
  (1) Ineligible publication types include reviews, meta-analyses, systematic reviews, conference abstracts, and nonclinical studies such as animal experiments. (2) Unavailability of data: incomplete raw data, inability to compute required effect estimates, or inability to obtain complete data after contacting the authors. (3) Duplicate publications or overlapping datasets from the same study.

### Data extraction and study quality assessment

Two reviewers (HW, ZJ) independently extracted data using a standardized Excel form, with cross-checking and resolution of discrepancies through discussion or by a third reviewer (LW). The extracted items included study characteristics (first author, publication year, country/region, study design, and sample size), baseline patient characteristics (age, sex, diabetes mellitus, hyperlipidemia, and smoking history), tear characteristics (anteroposterior tear length, mediolateral tear length, and tear size), muscle quality measures (Goutallier grade and global fatty degeneration index [GFDI]), outcomes (number of retears and total number of cases), and effect estimates (ORs and 95% CIs or data-enabling calculation).

Methodological quality was assessed for all included studies using the 11-item checklist recommended by the Agency for Healthcare Research and Quality (AHRQ) [26]. This tool evaluates study quality in domains including data sources, participant identification, quality assurance, and data reporting and handling. The total scores range from 0 to 11, with 0-3, 4-7, and 8-11 indicating low, moderate, and high quality, respectively. Two reviewers (HW, ZJ) independently assessed the quality of the studies. Disagreements were resolved through discussion and, when necessary, through consultation with a third reviewer (LW).

### Statistical analysis

For overall incidence, we extracted the number of retear events and the total sample size from each study and pooled these data using a single-arm proportion meta-analysis. For the binary outcome of retear compared with no retear, we used odds ratios with 95% CI as the effect measure to pool the associations between candidate risk factors and retear. Between-study heterogeneity was assessed using Cochran’s Q test and the I^2^ statistic. I^2^< 25% indicated low heterogeneity, 25-50% indicated moderate heterogeneity, and > 50% indicated high heterogeneity. A fixed-effects model was used when I^2^ was < 50% and *P* > 0.10; otherwise, a random-effects model was used. Sensitivity analyses were conducted using the leave-one-out approach, whereby the pooled estimate was recalculated after sequentially removing each study to determine whether the results changed materially, thereby assessing the robustness. For low-quality and high-risk-of-bias studies, additional sensitivity analyses were performed to assess their impact on the results. Publication bias was assessed by visual inspection of the funnel plot and quantitatively using Egger’s regression test; *P* < 0.05 suggested possible publication bias. All analyses were performed using STATA 14.0 (StataCorp, College Station, TX, USA) and RevMan 5.3 (Cochrane Collaboration, Oxford, UK). All tests were 2-sided, with α = 0.05.

## Results

This study performed quantitative syntheses to estimate the pooled overall retear rate and evaluate the potential risk factors for retear. The overall retear rate analysis included all studies with extractable numbers of retear events and total sample sizes, whereas the risk factor analyses included only cohort/case-control studies that reported ORs with 95% CIs or data enabling their calculation. Therefore, the number of studies included differed between the two syntheses.

### PRISMA Flow diagram

The search identified 1,013 records: 845 from WOS, 119 from the Cochrane Library, six from PubMed, and 43 from Embase. After removing 89 duplicates, 924 records underwent title and abstract screening, and 423 were excluded. During the full-text assessment, all studies reporting postoperative retear events and the total sample size were included in the single-arm proportion meta-analysis of the overall retear rate, yielding 273 studies. For the meta-analysis of risk factors, effect size information or raw data that could be used to calculate effect sizes were further extracted and cross-checked during the full-text assessment. Finally, 35 studies were included in the meta-analysis [6, 11, 16, 17, 27–35]. The study selection process is illustrated in Fig. 1.

### Study characteristics and risk-factor dataset

A single-arm proportion meta-analysis was performed on 273 studies, including 147,042 patients, with a mean patient age of 60.83 years (range, [52.09-70.45] years) across included studies. The baseline characteristics of all 273 studies included in the proportion meta-analysis are summarized in Supplementary Material 3. Thirty-five studies were included in the risk-factor meta-analyses (Table 1), comprising 12,031 patients (sample size per study, 86-1,000), with a mean age of 62.4 years (range, 54.7-78.3 years) and a mean follow-up duration of 13.8 months (range, 3-120 months). In total, 1,903 retears were reported, corresponding to an overall retear rate of 15.8% in the risk factor dataset. The evaluated risk factor domains were comprehensive and included comorbidities (e.g., diabetes mellitus and hyperlipidemia), lifestyle factors (smoking), tear size, and fatty infiltration.

**Table 1:** Baseline characteristics of included studies

Author (Year)	Country	Study design	Total sample size	Retears (*n*)	Healed (*n*)	Mean age (years)	Follow-up duration (months)
Yoon [27] 2024	Republic of Korea	Cohort study	135	31	104	63.13	6
Le [28] 2014	Australia	Cohort study	1000	174	826	59.01	6
Lee [29] 2024	Republic of Korea	Cohort study	723	151	572	62.4	3
Choi [30] 2014	Republic of Korea	Cohort study	147	25	122	62.8	23.4
Garcia [31] 2017	USA	Cohort study	86	21	65	62.1	6
Ohta [32] 2020	Japan	Cohort study	35	7	28	75.3	3
Lee [11] 2017	Republic of Korea	Cohort study	693	50	643	59.5	5.4
Duong [33] 2021	Australia	Cohort study	1962	271	1691	59	6
Aagaard [34] 2023	Sweden	Cohort study	57	21	36	61	15.75
Takeuchi [35] 2021	Japan	Cohort study	326	33	293	67	6
Iijima [65] 2019	Japan	Cohort study	107	32	75	67	12
Diebold [6] 2017	Australia	Cohort study	1600	212	1388	59	6
Yamakado [66] 2025	Japan	Cohort study	620	100	520	66.9	12
Daumillare [67] 2023	France	Cohort study	90	21	69	59.6	13.5
Harada [68] 2021	Japan	Cohort study	286	32	254	64.9	6
Shitara [69] 2024	Japan	Cohort study	143	29	114	70.45	24
Park [70] 2020	Republic of Korea	Cohort study	195	51	144	60.5	17.3
Kim [53] 2024	Republic of Korea	Case-control study	148	41	107	63.64	6
Kim [71] 2018	Republic of Korea	Cohort study	359	48	311	58.01	6
Barth [72] 2017	France	Cohort study	176	27	149	56	32.9
Chen [73] 2022	China	Cohort study	122	21	101	62.1	6
Cho [17] 2015	Republic of Korea	Cohort study	335	62	273	57.79	7.2
Takeda [74] 2024	Japan	Cohort study	370	71	299	62.59	4–6
Buyukdogan [75] 2021	Turkey	Cohort study	72	13	59	54.72	120
Shinohara [76] 2024	Japan	Cohort study	353	45	308	63.57	9.4
Kim KY [16] 2018	Republic of Korea	Cohort study	180	28	152	60.4	24
Ishitani [77] 2019	Japan	Cohort study	305	30	275	64.9	12
Cho [78] 2025	Republic of Korea	Cohort study	802	108	694	62.08	24
Son [79] 2024	Republic of Korea	Cohort study	73	10	63	62.4	12
Itoigawa [80] 2021	Japan	Case-control study	68	22	46	64.4	6
Cho [81] 2009	Republic of Korea	Cohort study	169	38	131	57.6	6
Cho [82] 2011	Republic of Korea	Cohort study	87	29	58	55.4	8.5
Kim [83] 2019	Republic of Korea	Cohort study	135	27	108	60.2	9
Bae [84] 2016	Republic of Korea	Cohort study	47	13	34	59.9	6
Park [85] 2016	Republic of Korea	Cohort study	25	9	16	78.3	12

### Methodological quality assessment of included studies

The methodological quality of all the included studies was assessed using the AHRQ checklist. The mean AHRQ score was 8.73 (SD = [0.83]), corresponding to an overall high-quality rating (scores 6-11), suggesting generally good methodological quality and supporting the credibility of the pooled estimates.

### Pooled effect estimates

#### Overall retear rate

A single-arm proportion meta-analysis of 273 studies (147,042 patients) demonstrated substantial heterogeneity (I^2^ = 88.28%, *P* < 0.001); therefore, a random-effects model was used. The pooled overall retear rate after ARCR in middle-aged and older patients was 17% (95% CI, 16%-18%).

#### Patient characteristics and lifestyle factors

##### Sex

**Fig. 2:**
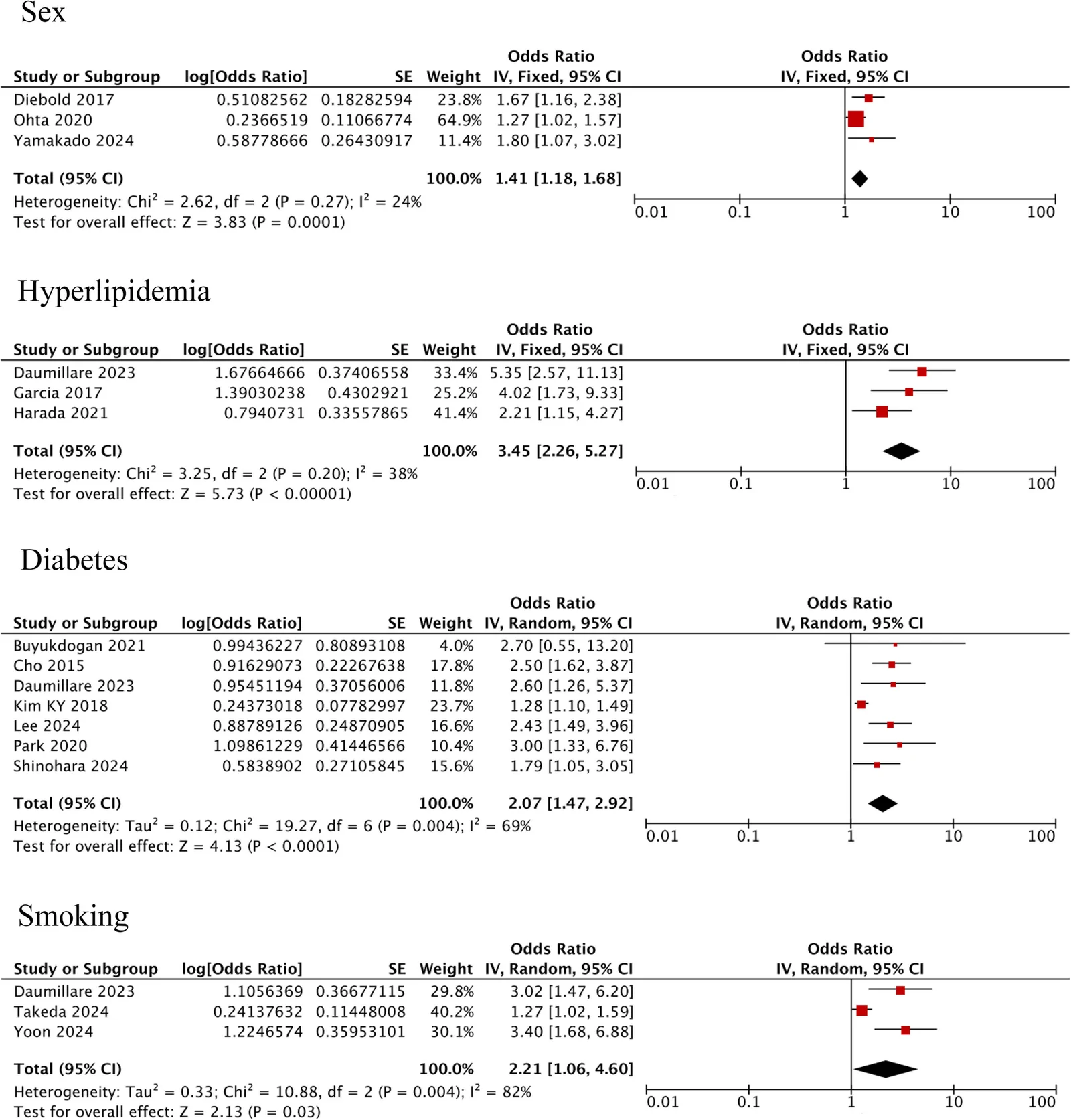
Forest plots of patient-related risk factors for rotator cuff retear

Three studies (1,441 patients) assessed the association between sex and rotator cuff retear. There was no significant heterogeneity (I^2^ = 24%, *P* = 0.27); therefore, a fixed-effects model was used. Male patients had a higher risk of retear than female patients (OR = 1.41, 95% CI, 1.18-1.68; *P* < 0.001) (Fig. 2).

##### Smoking history

Three studies (619 patients) reported the effect of smoking history on retear. Heterogeneity was high (I^2^ = 82%, *P* = 0.004) and a random-effects model was used. A history of smoking was associated with a significantly increased risk of retear (OR = 2.21, 95% CI, 1.06-4.60; *P* = 0.03) (Fig. 2).

#### Metabolic diseases

##### Diabetes mellitus

Seven studies (3,286 patients) evaluated diabetes mellitus and retear. Heterogeneity was high (I^2^ = 69%, *P* = 0.004) and a random-effects model was used. Diabetes mellitus was associated with a significantly increased risk of retear (OR = 2.07, 95% CI, 1.47-2.92; *P* < 0.001) (Fig. 2).

##### Hyperlipidemia

Three studies (1,177 patients) were included. Heterogeneity was moderate (I^2^ = 38%, *P* = 0.20) and a fixed-effects model was used. Hyperlipidemia was associated with a significantly increased risk of retear (OR = 3.45, 95% CI, 2.26-5.27; *P* < 0.00001) (Fig. 2).

#### Rotator cuff muscle fatty infiltration

Reports varied across the included studies, with some reporting Goutallier grades separately for individual muscles (most commonly the supraspinatus and infraspinatus), and others using the GFDI to summarize the overall rotator cuff degeneration. Rotator cuff muscle fatty infiltration was graded using the Goutallier classification [36]. As proposed by Goutallier et al., the GFDI was calculated as the mean Goutallier grade of the supraspinatus, infraspinatus, and subscapularis muscles.

##### Supraspinatus fatty infiltration

**Fig. 3:**
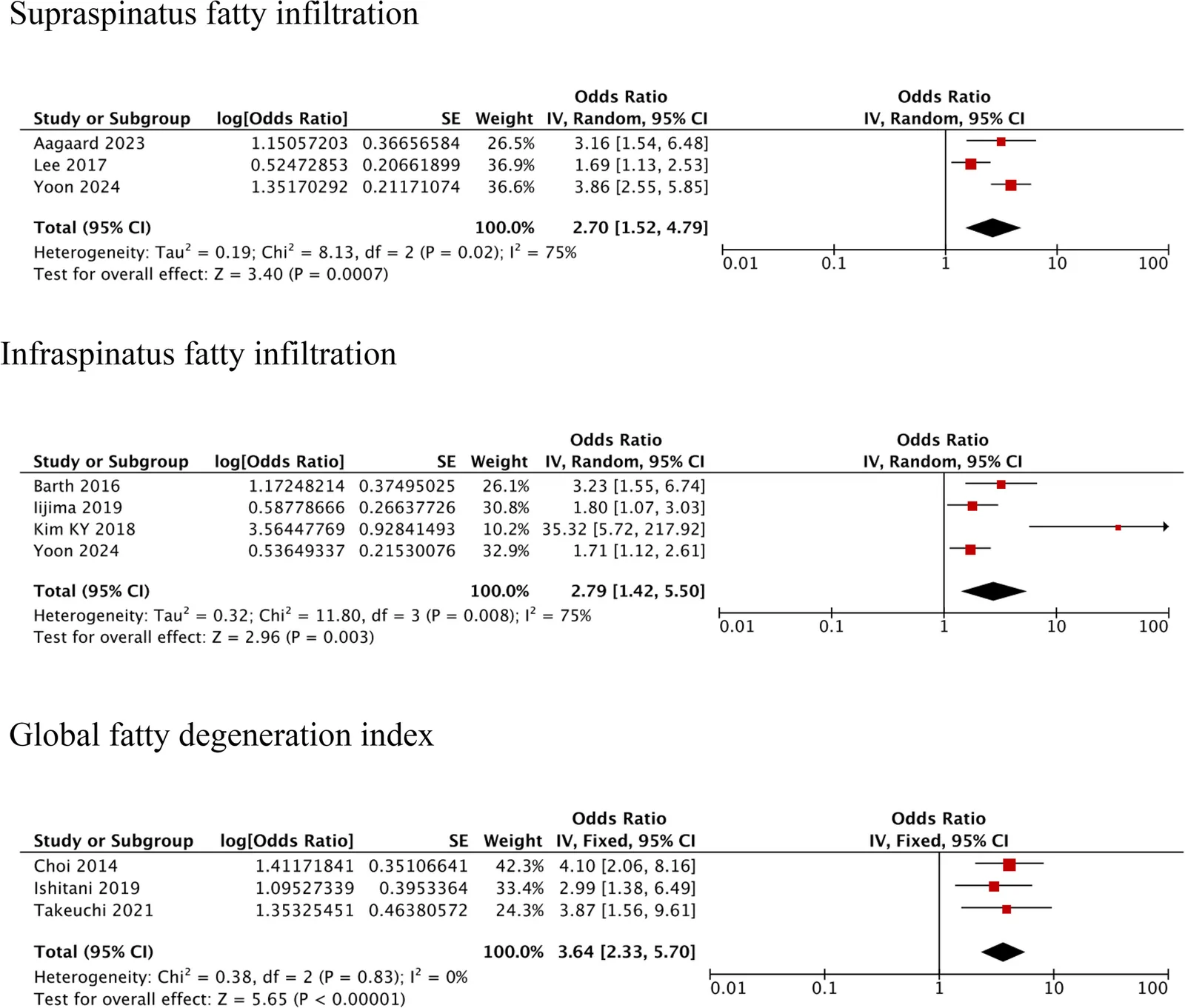
Forest plots of muscle fatty infiltration effects on rotator cuff retear

Three studies (710 patients) reported an association between supraspinatus Goutallier grade and retear risk. Heterogeneity was high (I^2^ = 75%, *P* = 0.02) and a random-effects model was used. Increasing supraspinatus fatty infiltration grade was associated with a significantly increased risk of retear (OR = 2.70, 95% CI, 1.52-4.79; *P* = 0.0007) (Fig. 3).

##### Infraspinatus fatty infiltration

Four studies (923 patients) reported an association between the infraspinatus Goutallier grade and retear. Heterogeneity was high (I^2^ = 75%, *P* = 0.008) and a random-effects model was used. Higher infraspinatus fatty infiltration was associated with a significantly increased risk of retear (OR = 2.79, 95% CI, 1.42-5.50; *P* = 0.003) (Fig. 3).

##### GFDI

Three studies (685 patients) evaluated the prognostic value of the GFDI. Heterogeneity was minimal (I^2^ = 0%, *P* = 0.83), and a fixed-effects model was used. As the GFDI increased from grade 0 to grade 4, the retear risk increased significantly (OR = 3.64, 95% CI, 2.33-5.70; *P* < 0.00001) (Fig. 3). Compared to single-muscle fatty infiltration measures, GFDI showed lower heterogeneity (I^2^ = 0% vs. 75%) and a larger effect estimate (OR = 3.64 vs. 2.70, 2.79), suggesting greater robustness and indicating that GFDI may be a more reliable predictor of postoperative retear than single-muscle assessment.

#### Tear-related characteristics

##### Anteroposterior tear length

**Fig. 4:**
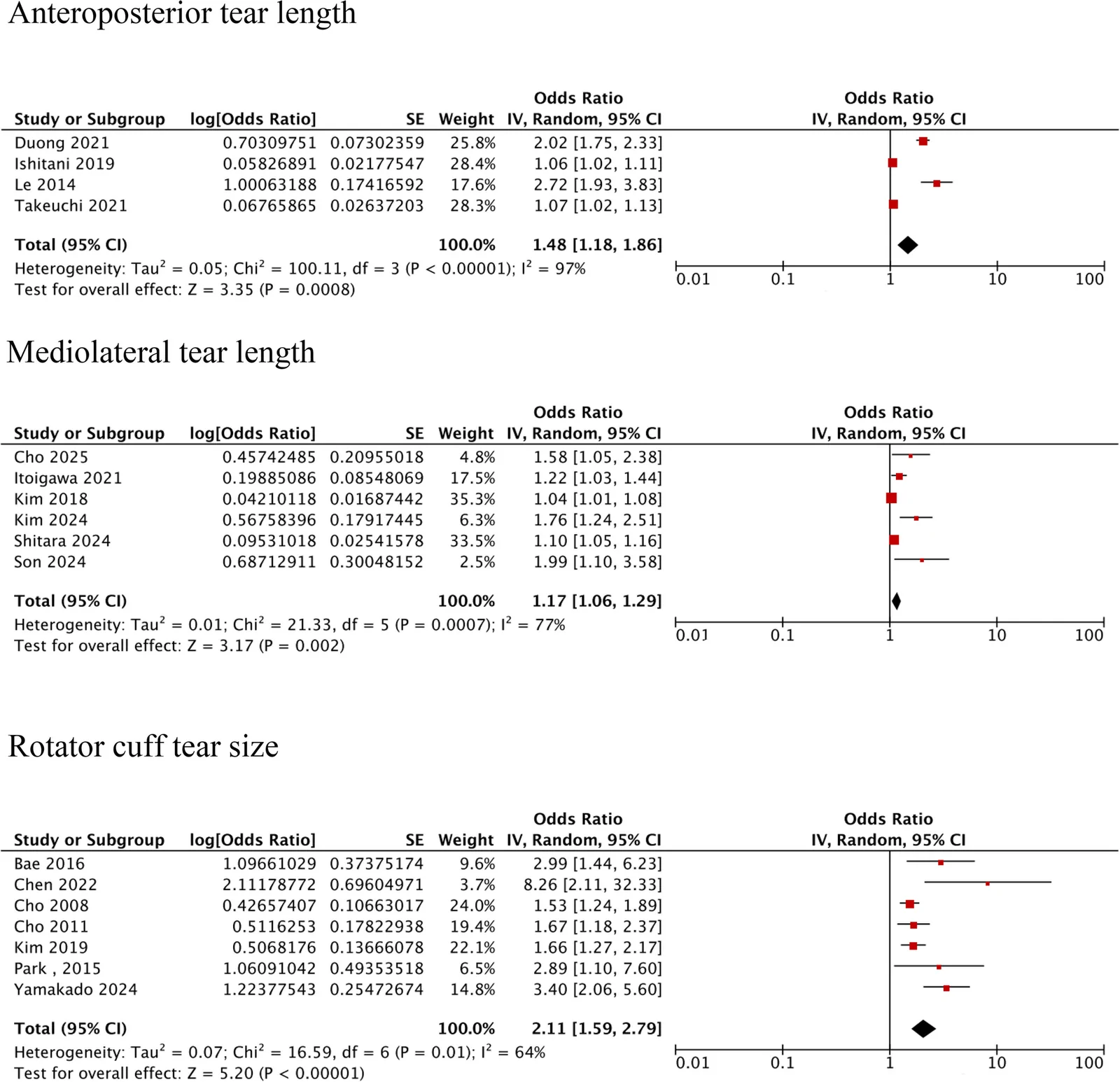
Forest plots of tear size effects on rotator cuff retear

Four studies (679 patients) reported the anteroposterior tear length. Heterogeneity was very high (I^2^ = 97%, *P* < 0.00001), and a random-effects model was used. Each 1-cm increase in anteroposterior tear length was associated with a 48% increase in retear risk (OR = 1.48, 95% CI, 1.18-1.86; *P* = 0.0008) (Fig. 4).

##### Mediolateral tear length

Six studies (1,301 patients) were included. Heterogeneity was high (I^2^ = 77%, *P* = 0.0007) and a random-effects model was used. Each 1-cm increase in mediolateral tear length was associated with a 17% increase in retear risk (OR = 1.17, 95% CI, 1.06-1.29; *P* = 0.002) (Fig. 4).

##### Tear size category

Some included studies reported tear size according to the DeOrio-Cofield classification [37], categorizing tears as small to medium (< 3 cm) versus large to massive (≥ 3 cm). Given that large and massive tears are independent risk factors for postoperative retear, this meta-analysis adopted the same cut-off value to compare the retear risk between these categories. Seven studies (2,154 patients) evaluated retear risk according to tear size category. Heterogeneity was high (I^2^ = 64%, *P* = 0.01) and a random-effects model was used. Large to massive tears were associated with a significantly increased risk of retear (OR = 2.11, 95% CI, 1.59–2.79; *P* < 0.00001) (Fig. 4).

### Sensitivity analysis and publication bias assessment

**Fig. 5:**
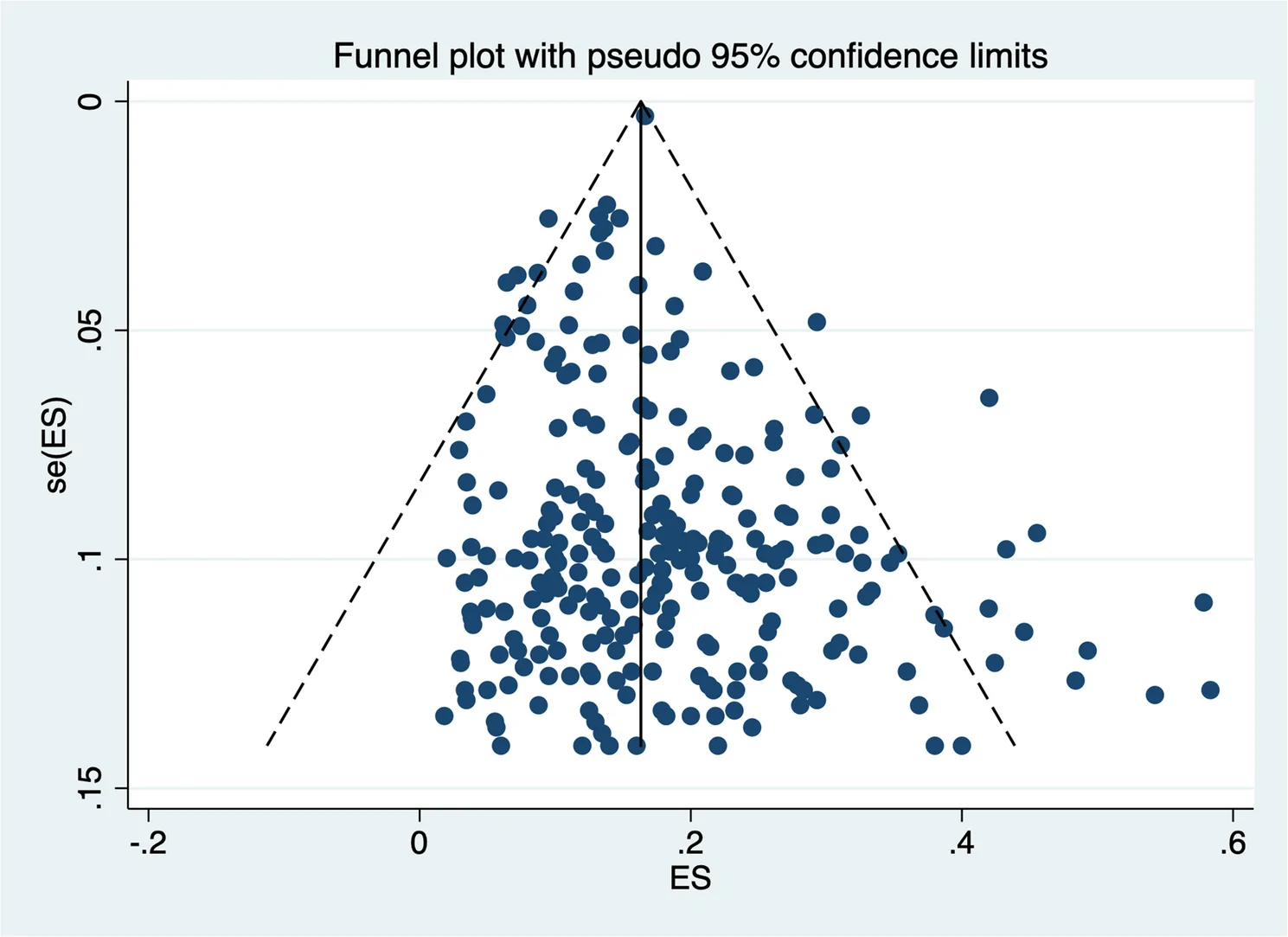
Funnel Plot of the Overall Retear Rate. Note: The vertical line indicates the pooled effect estimate, and the dashed lines represent pseudo 95% confidence limits. ES, effect size; se(ES), standard error of the effect size

Leave-one-out sensitivity analyses showed that the pooled estimates remained stable, with no single study exerting a decisive influence on the overall results, indicating good robustness. Publication bias was assessed in the 273 studies included in this single-arm proportion meta-analysis. Visual inspection of the funnel plot showed no clear asymmetry (Fig. 5). Egger’s regression test yielded an intercept (bias) of 0.140 (SE = 0.081), which was not statistically significant (t = 1.74, *P* = 0.084), and provided no clear evidence of linear asymmetry.

### Summary of key risk factors

Based on the meta-analysis results, the major risk factors for retear after ARCR in middle-aged and older patients are summarized in Table 2.

**Table 2:** Summary of key risk factors

Risk factor	Number of studies	Total sample size	Pooled OR (95% CI)	*P* value	I²
Male sex	3	1,441	1.41 (1.18–1.68)	< 0.001	24%
Diabetes mellitus	7	3,286	2.07 (1.47–2.92)	< 0.001	69%
Hyperlipidemia	3	1,177	3.45 (2.26–5.27)	< 0.00001	38%
Smoking history	3	619	2.21 (1.06–4.60)	0.03	82%
Supraspinatus fatty infiltration	3	710	2.70 (1.52–4.79)	0.0007	75%
Infraspinatus fatty infiltration	4	923	2.79 (1.42–5.50)	0.003	75%
GFDI	3	685	3.64 (2.33–5.70)	< 0.00001	0%
Anteroposterior tear length	4	679	1.48 (1.18–1.86)	0.0008	97%
Mediolateral tear length	6	1,301	1.17 (1.06–1.29)	0.002	77%
Large to massive tear	7	2,154	2.11 (1.59–2.79)	< 0.00001	64%

Fatty infiltration of the supraspinatus and infraspinatus muscles and GFDI were graded using the Goutallier classification. For anteroposterior and mediolateral tear length, the pooled ORs represent the effect per 1-cm increase. The comparison for large to massive tear (≥ 3 cm) was based on the DeOrio-Cofield classification, using small to medium (< 3 cm) as the reference category

## Discussion

### Main findings and clinical implications

To the best of our knowledge, few prior studies have pooled the overall retear incidence after ARCR in middle-aged and older patients using a single-arm proportion meta-analysis. In the present review, 273 studies comprising 147,042 patients were included to estimate incidence, yielding a pooled postoperative retear rate of 17%, which may serve as a benchmark for this population. In addition, 35 cohort and case-control studies (12,031 patients) reported ORs or provided data enabling their calculation and were therefore included in the risk-factor meta-analysis to quantify associations between potential risk factors and retear. Notably, the retear rate within the risk factor dataset (15.8%) was close to the pooled overall retear rate (17%), suggesting consistency between the 2 quantitative syntheses for estimating the level of retear occurrence.

The results indicated that metabolic factors, including hyperlipidemia and diabetes mellitus, and smoking history, were significantly associated with an increased risk of retear. Regarding patient characteristics, male sex may be a risk factor for retear, potentially because of the greater physical workload and more frequent use of the upper extremities, leading to increased postoperative shoulder loading. For tear characteristics, each 1-cm increase in the anteroposterior tear length increased the retear risk by 48%, and each 1-cm increase in the mediolateral tear length increased the retear risk by 17%, indicating a higher retear risk with larger tears. In terms of muscle quality, supraspinatus fatty infiltration, infraspinatus fatty infiltration, and the GFDI were important predictors of retear, with the GFDI showing a particularly strong prognostic value.

Although ARCR generally yields satisfactory clinical outcomes, repair of the fibrocartilaginous tendon-to-bone interface cannot restore the native 4-layer enthesis structure and instead forms Sharpey-like fibers that attach the tendon to the bone [38–40]. In middle-aged and older patients, the deterioration of tendon matrix function, local metabolic disturbances, disorganized collagen fiber architecture, and limited intrinsic healing capacity may contribute to retear risk [41]. Further improvement in the mechanical and histological qualities of the tendon-to-bone interface to enable faster and safer functional recovery remains a key clinical challenge. The 2025 American Academy of Orthopaedic Surgeons (AAOS) updated clinical practice guideline on rotator cuff injuries emphasizes the impact of comorbidities such as diabetes mellitus, hypertension, hypercholesterolemia, smoking, and body mass index on rotator cuff outcomes [42]. By quantitatively synthesizing retear-related risk factors across metabolic status, patient characteristics, tear morphology, and muscle quality, this study provides evidence to support preoperative risk stratification, individualized treatment decisions, and postoperative management. Compared with previous single-center, small-sample studies, the present study included a larger sample size, broader geographic representation, and a more comprehensive set of risk factors, enhancing generalizability.

### Pathophysiologic interpretation of risk factors

#### Metabolic abnormalities and impaired tendon-to-bone healing

A substantial body of basic and clinical research suggests that abnormal glucose metabolism can impair tendon-to-bone healing via multiple pathways [43]. Animal studies have shown that after rotator cuff repair in diabetic rats, the tendon-to-bone interface healing strength is appropriately 44% of that in non-diabetic controls [44]. Accumulation of advanced glycation end products (AGEs) in the supraspinatus muscle and healing tendon in diabetic models can cause abnormal collagen cross-linking and impaired cell–matrix interactions, thereby weakening biomechanical properties and histologic reconstruction at the tendon-to-bone interface [44]. Furthermore, hyperglycemia can upregulate inflammatory mediators such as matrix metalloproteinase-9 (MMP-9) and interleukin-6 (IL-6), disrupting the balance between extracellular matrix synthesis and degradation and further increasing the risk of healing failure after rotator cuff repair [45]. In the present meta-analysis, diabetes mellitus was associated with an 2.07-fold increased risk of retear in middle-aged and older patients, consistent with prior biomechanical and molecular mechanistic evidence and underscoring the role of dysglycemia in retear [44–46]. Clinically, the metabolic status should be carefully evaluated in patients with diabetes mellitus, with appropriate timing and selection of the surgical approach and strict perioperative glycemic control to improve tendon-to-bone healing and reduce retear risk.

Hyperlipidemia may also interfere with tendon-to-bone healing through multiple mechanisms. Animal studies indicate that hyperlipidemia can exacerbate supraspinatus fatty infiltration and disrupt the tendon-to-bone interface, leading to disorganized collagen fiber arrangement and looser tissue structure, with biomechanical properties substantially lower than in controls [47]. A rat rotator cuff repair model likewise demonstrated that hypercholesterolemia significantly reduced normalized stiffness at the tendon-to-bone interface at 4 weeks postoperatively [48]. In this large-sample meta-analysis, hyperlipidemia was identified as an independent risk factor for retear in middle-aged and older patients (OR = 3.45), with an effect size larger than that of diabetes mellitus, highlighting the potential importance of lipid dysregulation in repair failure. Standardized perioperative lipid-lowering and metabolic management in patients with hyperlipidemia may help mitigate fatty infiltration of the rotator cuff muscle, improve the local microenvironment at the tendon-to-bone interface, and provide potential targets for interventions to enhance healing.

Smoking is another modifiable risk factor that impairs rotator cuff healing. Basic studies indicate that nicotine exposure delays tendon-to-bone healing in rat rotator cuff repair models, characterized by compromised blood supply at the interface, decreased osteogenic/tenogenic cellular activity, and insufficient mechanical strength [49]. In this study, a history of smoking was associated with an 2.21-fold increased risk of retear. Although the heterogeneity was high (I^2^ = 82%), the overall direction of the effect was consistent with previous conclusions that smoking adversely affects healing after ARCR, suggesting a clear negative impact [50]. Therefore, patients with a history of smoking should be advised to stop smoking at least 6 months preoperatively and throughout the perioperative period to optimize the healing microenvironment and reduce the risks of infection and repair failure, thereby improving the long-term success of rotator cuff repair [51]. 

Therefore, the role of metabolic management in rotator cuff repair should be emphasized in clinical practice. For patients with established diabetes mellitus or hyperlipidemia, the optimization of blood glucose and lipid levels before elective surgery may provide a more favorable systemic metabolic environment for repair. The first 3–6 months postoperatively represent a critical period for tendon-to-bone healing, and metabolic homeostasis should be monitored and maintained during this period [52]. Patients with diabetes mellitus should intensify glucose monitoring and avoid marked glycemic fluctuations, whereas those with hyperlipidemia should continue regular lipid-lowering therapy to prevent recurrent lipid elevation from adversely affecting healing [53, 54]. In addition, perioperative nutritional support should be emphasized, with appropriate supplementation of vitamin D, calcium, and high-quality proteins to support bone and tendon health [55]. 

#### Tear size and biomechanical imbalance

Tear size is a key determinant of the prognosis after rotator cuff repair. Prior studies have indicated that larger tears are associated with lower healing rates and higher retear rates, which may be related to increased repair tension and technical difficulties. [56] Large tears often involve multiple tendons with substantial degeneration and retraction [57]. Tokunaga et al. [56] found that larger tears exert greater mechanical stress on the tendon and anchor points during healing, which may increase the risk of failed tendon-to-bone ingrowth. In this study, each 1-cm increase in anteroposterior tear length increased the retear risk by 48%, and each 1-cm increase in mediolateral tear length increased the retear risk by 17%. These findings provide quantitative evidence supporting precise preoperative measurement of tear size, risk assessment, and individualized surgical planning. For high-risk patients with massive or extensive tears and severe degeneration and retraction, biological augmentation (e.g., patch augmentation or platelet-rich plasma) may be considered in addition to standard repair to improve local mechanical and biological conditions and potentially reduce postoperative retear rates.

#### Muscle fatty infiltration

Rotator cuff tears are frequently accompanied by varying degrees of fatty infiltration, which degrade muscle quality and have been consistently associated with poorer structural healing after repair [58]. Accordingly, interventions aimed at preventing or reversing rotator cuff muscle degeneration may improve longer-term outcomes following repair. Fatty infiltration is most commonly graded using the Goutallier classification (grades 0–4), and prior studies have reported substantially higher postoperative retear rates when preoperative fatty infiltration is grade > 2 [59]. The pathophysiology is closely linked to abnormal accumulation and differentiation of fibro-adipogenic progenitor cells within muscle: on the one hand, their accumulation after rotator cuff injury provides a key substrate for fatty infiltration [60]; on the other hand, multiple signaling pathways regulate adipogenic differentiation, including reduced inhibition due to Wnt pathway inactivation [61], increased adipogenesis after suppression of cilia-mediated Hedgehog signaling [62], promotion of adipogenesis through Notch signaling [63], potentially in synergy with tumor necrosis factor-α (TNF-α), and regulation via the bone morphogenetic protein/transforming growth factor-β (BMP/TGF-β) axis, with downstream effects on matrix metalloproteinase-13 (MMP-13) [64]. 

This study further evaluated the prognostic value of the GFDI, which reflects overall fatty infiltration, by averaging the Goutallier grades of the supraspinatus, infraspinatus, and subscapularis. As GFDI increased, retear risk increased 3.64-fold (95% CI, 2.33–5.70), with minimal heterogeneity (I^2^ = 0%), indicating substantially greater robustness than single-muscle fatty infiltration assessments (supraspinatus I^2^ = 75%; infraspinatus I^2^ =75%).

### Study limitations

This study had several limitations. First, follow-up duration varied widely across included studies (3-120 months). Studies with shorter follow-up periods may underestimate retear rates by failing to capture late-occurring retears, whereas longer follow-ups may be more susceptible to degenerative changes and attrition bias. We therefore retained all studies but acknowledge this as a potential source of time-window bias. Future meta-analyses restricted to studies with a minimum follow-up of 1–2 years postoperatively are warranted to provide more temporally robust estimates. Second, several potentially important risk factors, including cuff tear arthropathy and acromiohumeral distance narrowing, were not meta-analyzed, owing to an insufficient number of studies reporting these variables in an extractable format. As new studies become available, we will update this meta-analysis to incorporate these structural factors and provide more comprehensive risk stratification. Lastly, while conclusions regarding the major risk factors appear relatively robust, findings for factors supported by a limited number of studies require further validation through high-quality prospective investigations.

## Conclusion

This systematic review and meta-analysis indicated that the overall retear rate after ARCR in middle-aged and older patients was 17%. Risk factor meta-analyses further suggested that retear is closely associated with several identifiable factors. Hyperlipidemia, diabetes mellitus, male sex, smoking, larger tear size, and greater fatty infiltration of the rotator cuff muscle were all associated with a significantly increased risk of retear. These findings support the incorporation of the management of metabolic comorbidities and accurate preoperative assessment of tear size and muscle quality into routine decision-making to guide surgical approach selection.

## Supplementary Information

Supplementary Material 1.

Supplementary Material 2.

Supplementary Material 3.

## Data Availability

The datasets used during the present study are available from the corresponding author on reasonable request.
