# Supplementary material for: Incidence and risk factors for rotator cuff retear after arthroscopic rotator cuff repair in middle-aged and older patients: a systematic review and meta-analysis
Source: BMC Musculoskelet Disord. 2026 Aug 10;27:958. doi: 10.1186/s12891-026-10337-7 (PMC13737563; doi:10.1186/s12891-026-10337-7)
Supplement: Supplementary file 2 [file 12891_2026_10337_MOESM2_ESM.docx]

Supplementary Material 2. Full electronic search strategies

2.1 Search strategy for Web of Science

1:Cuff Injury, Rotator (Topic) or Injury, Rotator Cuff (Topic) or Rotator Cuff Injury (Topic) or Rotator Cuff Tears (Topic) or Rotator Cuff Tear (Topic) or Tear, Rotator Cuff (Topic) or Tears, Rotator Cuff (Topic) or Rotator Cuff Injuries (Topic) and Preprint Citation Index (Exclude – Database) and Research Commons (Exclude – Database)

2:Arthroscopies (Topic) or Arthroscopic Surgical Procedures (Topic) or Arthroscopic Surgical Procedure (Topic) or Procedure, Arthroscopic Surgical (Topic) or Procedures, Arthroscopic Surgical (Topic) or Surgical Procedure, Arthroscopic (Topic) or Arthroscopic Surgery (Topic) or Arthroscopic Surgeries (Topic) or Surgeries, Arthroscopic (Topic) or Surgery, Arthroscopic (Topic) or Surgical Procedures, Arthroscopic (Topic) and Preprint Citation Index (Exclude – Database) and Research Commons (Exclude – Database)

3:elderly (Topic) or aged (Topic) or Middle Aged (Topic) and Preprint Citation Index (Exclude – Database) and Research Commons (Exclude – Database)

4:retear (Topic) or rotator cuff retear (Topic) or Revision (Topic) and Preprint Citation Index (Exclude – Database) and Research Commons (Exclude – Database)

#1 AND #2 AND #3 AND #4 and Preprint Citation Index (Exclude – Database) and Research Commons (Exclude – Database)

The search covered inception through October 1, 2025, with no language restrictions.

2.2 Search strategy for Cochrane CENTRAL

1:(Cuff Injury, Rotator):ti,ab,kw OR (Injury, Rotator Cuff):ti,ab,kw OR (Rotator Cuff Injury):ti,ab,kw OR (Rotator Cuff Tears):ti,ab,kw OR (Rotator Cuff Tear):ti,ab,kw OR (Tear, Rotator Cuff):ti,ab,kw OR (Tears, Rotator Cuff):ti,ab,kw OR (rotator cuff rupture):ti,ab,kw OR (Rotator Cuff Injuries):ti,ab,kw

2:(Arthroscopies):ti,ab,kw OR (Arthroscopic Surgical Procedures):ti,ab,kw OR (Arthroscopic Surgical Procedure):ti,ab,kw OR (Procedure, Arthroscopic Surgical):ti,ab,kw OR (Procedures, Arthroscopic Surgical):ti,ab,kw OR (Surgical Procedure, Arthroscopic):ti,ab,kw OR (Arthroscopic Surgery):ti,ab,kw OR (Arthroscopic Surgeries):ti,ab,kw OR (Surgeries, Arthroscopic):ti,ab,kw OR (Surgery, Arthroscopic):ti,ab,kw OR (Surgical Procedures, Arthroscopic):ti,ab,kw OR (arthroscopic rotator cuff repair):ti,ab,kw

3:(elderly):ti,ab,kw OR (aged):ti,ab,kw OR (Middle Aged):ti,ab,kw

4:retear OR rotator cuff retear OR Revision

#1 AND #2 AND #3 AND #4

The search covered inception through October 1, 2025, with no language restrictions.

2.3 Search strategy for PubMed

((((((((((((((Arthroscopy[Title/Abstract]) OR (Arthroscopies[Title/Abstract])) OR (Arthroscopic Surgical Procedures[Title/Abstract])) OR (Arthroscopic Surgical Procedure[Title/Abstract])) OR (Procedure, Arthroscopic Surgical[Title/Abstract])) OR (Procedures, Arthroscopic Surgical[Title/Abstract])) OR (Surgical Procedure, Arthroscopic[Title/Abstract])) OR (Arthroscopic Surgery[Title/Abstract])) OR (Arthroscopic Surgeries[Title/Abstract])) OR (Surgeries, Arthroscopic[Title/Abstract])) OR (Surgery, Arthroscopic[Title/Abstract])) OR (Surgical Procedures, Arthroscopic[Title/Abstract])) AND ((((((((Rotator Cuff Injuries[Title/Abstract]) OR (Cuff Injury, Rotator[Title/Abstract])) OR (Injury, Rotator Cuff[Title/Abstract])) OR (Rotator Cuff Injury[Title/Abstract])) OR (Rotator Cuff Tears[Title/Abstract])) OR (Rotator Cuff Tear[Title/Abstract])) OR (Tear, Rotator Cuff[Title/Abstract])) OR (Tears, Rotator Cuff[Title/Abstract]))) AND ((((elderly[Title/Abstract]) OR (aged[Title/Abstract])) OR (Middle Age[Title/Abstract])) OR (Middle Aged[Title/Abstract]))) AND (retear)

The search covered inception through October 1, 2025, with no language restrictions.

2.4 Search strategy for Embase

1：'Cuff Injury, Rotator':ab,ti or 'Injury, Rotator Cuff':ab,ti or 'Rotator Cuff Injury':ab,ti or 'Rotator Cuff Tears':ab,ti or 'Rotator Cuff Tear':ab,ti or 'Tear, Rotator Cuff':ab,ti or 'Tears, Rotator Cuff':ab,ti or 'rotator cuff rupture':ab,ti or 'Rotator Cuff Injuries':ab,ti

2: 'Arthroscopies':ab,ti or 'Arthroscopic Surgical Procedures':ab,ti or 'Arthroscopic Surgical Procedure':ab,ti or 'Procedure, Arthroscopic Surgical':ab,ti or 'Procedures, Arthroscopic Surgical':ab,ti or 'Surgical Procedure, Arthroscopic':ab,ti or 'Arthroscopic Surgery':ab,ti or 'Arthroscopic Surgeries':ab,ti or 'Surgeries, Arthroscopic':ab,ti or 'Surgery, Arthroscopic':ab,ti or 'Surgical Procedures, Arthroscopic':ab,ti or 'arthroscopic rotator cuff repair':ab,ti

3: 'elderly':ab,ti or 'aged':ab,ti or 'Middle Aged':ab,ti

4: retear OR rotator cuff retear OR Revision

#1 AND #2 AND #3 AND #4

The search covered inception through October 1, 2025, with no language restrictions.
